# Questions to guide cancer evolution as a framework for furthering progress in cancer research and sustainable patient outcomes

**DOI:** 10.1007/s12032-022-01721-z

**Published:** 2022-07-04

**Authors:** Jason A. Somarelli, James DeGregori, Marco Gerlinger, Henry H. Heng, Andriy Marusyk, Danny R. Welch, Frank H. Laukien

**Affiliations:** 1 Department of Medicine, Duke Cancer Institute, Duke University Medical Center, Durham, NC USA; 2 Department of Biochemistry and Molecular Genetics, School of Medicine, University of Colorado Anschutz Medical Campus, Aurora, CO USA; 3 Barts Cancer Institute, Queen Mary University of London, London, UK; 4 St Bartholomew’s Hospital Cancer Centre, London, UK; 5 Center for Molecular Medicine and Genetics, Department of Pathology, Wayne State University School of Medicine, Detroit, MI 48201 USA; 6 Department of Cancer Physiology, Moffitt Cancer Center, Tampa, FL 33612 USA; 7 Department of Cancer Biology, The University of Kansas Medical Center and The University of Kansas Cancer Center, Kansas City, KS USA; 8 Department of Chemistry & Chemical Biology, Harvard University, Cambridge, MA USA

**Keywords:** Cancer prevention, Cancer diagnosis, Cancer treatment, Cancer ecology, Evolutionary fitness landscapes

## Abstract

We appear to be faced with ‘two truths’ in cancer—one of major advances and successes and another one of remaining short-comings and significant challenges. Despite decades of research and substantial progress in treating cancer, most patients with metastatic cancer still experience great suffering and poor outcomes. Metastatic cancer, for the vast majority of patients, remains incurable. In the context of advanced disease, many clinical trials report only incremental advances in progression-free and overall survival. At the same time, the breadth and depth of new scientific discoveries in cancer research are staggering. These discoveries are providing increasing mechanistic detail into the inner workings of normal and cancer cells, as well as into cancer–host interactions; however, progress remains frustratingly slow in translating these discoveries into improved diagnostic, prognostic, and therapeutic interventions. Despite enormous advances in cancer research and progress in progression-free survival, or even cures, for certain cancer types—with earlier detection followed by surgical, adjuvant, targeted, or immuno- therapies, we must challenge ourselves to do even better where patients do not respond or experience evolving therapy resistance. We propose that defining cancer evolution as a separate domain of study and integrating the concept of evolvability as a core hallmark of cancer can help position scientific discoveries into a framework that can be more effectively harnessed to improve cancer detection and therapy outcomes and to eventually decrease cancer lethality. In this perspective, we present key questions and suggested areas of study that must be considered—not only by the field of cancer evolution, but by all investigators researching, diagnosing, and treating cancer.

## Remarkable successes and continued failings in cancer research and patient care

Decades of research has led to substantial progress in diagnosing and treating cancer. For instance, cancer death rates in the United States have fallen steadily from their peaks in the 1990s, largely due to improvements in early detection, smoking cessation, and new treatment paradigms [1]. Among the many examples, transformational treatment progress has been made in multiple malignancies, such as breast cancer [2], melanoma [3], childhood acute lymphoblastic leukemia [4], bladder cancer [5], renal cell carcinoma [6], prostate cancer [7], and lung cancer [8], among others, with early detection, the advent of immunotherapy [9], and a better genetic understanding of oncogenic drivers that has enabled the effective use of targeted therapies.

Despite this progress, however, global cancer death rates are more varied by country, with death rates in some countries even increasing [10] and substantial disparities in cancer outcomes by race [11] and socioeconomic status [12]. In fact, ten million people around the world still die from cancer every year [13]. It is clear that our significant progress and successes are not sufficient, and a continued and urgent need remains to further improve cancer prevention, detection, and treatment. In particular, we are faced with the clinical reality that too many cancer patients, even if they initially respond well to treatment, often later succumb due to the evolution of metastatic disease and therapy resistance.

The improvements we see in cancer outcomes are the result of transdisciplinary efforts from researchers and physicians working across numerous fields. This research effort has led to an unprecedented number of discoveries using in vitro systems, mouse models, and patient genomics studies. These discoveries are accelerating insights into the mechanisms of action and regulation of cellular function for both normal and neoplastic cells. Yet, the vast majority of discoveries are not being fully exploited for more *sustainable* enhancements in quality and quantity of life for cancer patients. There remain substantial barriers to effectively translate the growing knowledge of cellular function into improved outcomes for cancer patients, and there are major areas of molecular, cellular, and tissue systems biology that are only beginning to be explored, as new multi-omics, single-cell and spatial biology technologies become more widely available.

We believe that integration of evolutionary concepts into cancer research and treatment can improve the status quo and provide a framework for scientific advances to be more effectively leveraged to make cancer more manageable, perhaps chronic in some cases, significantly increase progression-free survival further, and decrease overall cancer lethality [14, 15]. In fact, we propose that the real-time evolutionary dynamics of cancer cell populations must be considered a foundational hallmark of cancer: one that has been described for decades [16, 17], but so far has been under-appreciated.

## Key questions for the cancer research community at the intersection of cancer and evolution

How will the field of Cancer Evolution help to drive change in the fields of cancer prevention, early detection, diagnosis, and treatment? The change we seek will be made by attacking questions and controversies head on. Key scientific questions and medical controversies need urgent further research without preconceptions and stigma that could impede the needed faster progress in cancer biology, diagnostics, and therapy. Questions need to be stated clearly, without implied reproach, and agnostic as to outcome, as present and future patient longevity and quality of life depend on achieving deeper insights and greater clarity. To this end, we present below several key questions and areas of study to consider—not only by the field of Cancer Evolution, but also by all investigators and oncologists studying and treating cancer.

1. Can we make sense of cancer evolution primarily by analyses of genetic diversity alone, such as mutational signatures, copy number alterations, etc.? In what contexts can cancer evolution be reduced to the study of driver mutations? Do we need to explicitly consider other processes, such as massive diversification due to the genome chaos created by genomic instability and smaller scale mutational diversification? In what contexts is cancer progressing primarily through successive mutation microevolution or dominated by more punctuated genetic and genomic macroevolution with major genome rearrangements or ‘genome chaos’? Do analyses of genetics require a consideration of all sources of molecular diversification of heritable variability, including adaptive diversification through both mutational- and plasticity-mediated mechanisms and context-specific selection forces?
2. How do we effectively combine the more reductionist studies with systems-level approaches to identify novel prognostic, predictive, and durable therapeutic strategies? How important and actionable is it to characterize the tissue and tumor microenvironments in conjunction with genetic/karyotype, epigenetic, and transcriptomic changes for the development of improved cancer biomarkers and therapies?
3. Is a broader genomics perspective, encompassing genetics, epigenetics, transcriptomics, and karyotype/aneuploidy analysis sufficient to understand cancer cell evolution? To what extent are genetic mutations or genomic changes buffered or purged by the cell, tissue, or organismal physiology? What other layers of cellular phenotype and regulation, such as proteomics, post-translational modifications, protein–protein interactions, and metabolomics are required to more fully understand the evolutionary processes that underlie oncogenesis, progression, therapy resistance, and metastasis?
4. Is cancer primarily a disease of rogue, plastic, fast-evolving cancer cells that acquire increasing evolvability and therapy resistance? Or is cancer also a tissue and organismal disease, where tissue control of cancerous cells has failed? Can this tissue control of cancer be potentially modulated or restored and can this reveal new strategies for cancer prevention or therapy? How can we effectively leverage interactions between cancer cells and non-cancer cells (e.g., immune cells or stromal cells) in the tumor microenvironment? How prevalent is reprogramming of the tumor microenvironment and even the entire immune system and distant organs by cancer?
5. What more is needed to complement existing genetics / genomics data in understanding the creation of metastatic niches, metastatic spread, and the vexing, but pervasive evolution of therapy resistance? Are there insufficient studies of paired primary and metastasis samples to infer processes that underpin the metastatic cascade? Can the karyotype profile provide a prediction based on how genome-level changes shape the macroevolution of cancer, while gene mutations promote microevolution [18]? How can we further capitalize on the study of circulating factors that portend poor clinical outcomes, such as circulating cell-free biomolecules, extracellular vesicles, and circulating tumor cells, to improve prediction and prevention of metastasis?
6. How can we improve current standard models for cancer? With what organoid or other human-like models can this translation paradigm be enhanced? How can insights from in vitro models be utilized more effectively to rapidly advance progress in clinical oncology? What new experimental models of evolution need to be developed for addressing evolutionary trajectories and cancer evolvability? To what extent can bioinformatics, mathematical, and other in silico approaches replace or augment cancer model systems and at what point are translational studies in patient samples required?
7. When do aggressive chemo-, targeted, or immuno-therapies—when they fall short of cure—lead to accelerating cancer evolution and therapy resistance [19]? What new therapeutic strategies can be employed to slow cancer evolution and contribute to long-term extensions of patient lives? Are chemo-, targeted, or immune-oncology monotherapies, which continue until clinical progression resumes, suboptimal or ill-advised as cancer extinction cures? Which evolutionary phases, from punctuated macroevolution to stepwise Darwinian microevolution, should be targeted for a better treatment outcome [20]? How do we balance small increases in progression-free survival with the risk of accelerating disease in others via active cell biology feedback mechanisms, such as stress-induced mutagenesis?

## Conclusions

Modern anti-cancer therapies have increased survival in many patients, and in a minority of patients have even led to cures. Solid neoplasms can be resected or ablated by radiation in early stages, and when combined with ‘extinction cures’ through therapeutic adjuvant treatment, these strategies have significantly increased survival rates in many solid tumors.

However, we must also dare to ask why we are not doing better in achieving cure and reducing cancer developing in the first place. Studies show how aggressive strategies can trigger active cell biology resistance mechanisms that enable therapy tolerance, enhance cancer evolvability, and ultimately accelerate cancer clonal evolution, and metastasis—but we still do not understand the generality of these mechanisms nor how to evade them. We need to better understand how and when treatment additionally promotes selection for new phenotypes that drive drug-resistant relapses or even new cancers. We need to fully appreciate the extent to which maximally tolerated dose monotherapies inadvertently trigger and accelerate cancer progression and metastasis of certain cancer patients [21].

The two truths in cancer are that we should celebrate our many advances in the understanding of disease biology and treatment successes, *and* that we simultaneously must challenge ourselves to do better. The field of cancer evolution has the potential to contribute to the paradigm shift from often not sustainable advances in patient care to hopefully more disruptive leaps in understanding cancer prevention, diagnosis, and treatment for major increases in progression-free survival, for improvements in quality of life, and ultimately, to reduce this disease to a chronic and manageable form, or to offer more cures.
